# Pfizer-BioNTech and Sinopharm: A Comparative Study on Post-Vaccination Antibody Titers

**DOI:** 10.3390/vaccines9111223

**Published:** 2021-10-21

**Authors:** Rami Alqassieh, Aiman Suleiman, Sami Abu-Halaweh, Abeer Santarisi, Omar Shatnawi, Lara Shdaifat, Amjed Tarifi, Mohammad Al-Tamimi, Abdel-Ellah Al-Shudifat, Heba Alsmadi, Ahmed Al Sharqawi, Hadeel Alnawaiseh, Yara Anasweh, Farah Abo Domaidah, Haneen Abu Jaber, Mohammad Rashid Al-Zarir, Isam Bsisu

**Affiliations:** 1 Department of General and Specialized Surgery, Faculty of Medicine, The Hashemite University, Zarqa 13133, Jordan; Rami_qaisieh@yahoo.com (R.A.); amjed.tarifi@gmail.com (A.T.); 2 Beth Israel Deaconess Medical Center, Anesthesia and Intensive Care Department, Harvard Medical School, Boston, MA 02215, USA; Asuleima@bidmc.harvard.edu; 3 Department of Anesthesia and Intensive Care, School of Medicine, The University of Jordan, Amman 11942, Jordan; s.halaweh@ju.edu.jo (S.A.-H.); Sw006999@gmail.com (H.A.); sharqawiahmed@gmail.com (A.A.S.); Hanabrklna@gmail.com (H.A.); Yara.anasweh@gmail.com (Y.A.); farahabodomaidah@gmail.com (F.A.D.); abujaber.haneen@gmail.com (H.A.J.); rashed.zaareer@gmail.com (M.R.A.-Z.); 4 Department of Emergency Medicine and Accidents, School of Medicine, The University of Jordan, Amman 11942, Jordan; abeer_santarisi@yahoo.com; 5 Department of Cancer Biology, Wake Forest School of Medicine and Wake Forest Baptist Health, Winston-Salem, NC 27157, USA; Omar.juve10@gmail.com; 6 School of Medicine, Mutah University, Karak 61710, Jordan; Shdaifatlara@gmail.com; 7 Faculty of Medicine, The Hashemite University, Zarqa 13133, Jordan; mohammad.altamimi@hu.edu.jo (M.A.-T.); amalmufleh@hotmail.com (A.-E.A.-S.)

**Keywords:** COVID-19, vaccine, Pfizer-BioNTech, Sinopharm, antibody, titers

## Abstract

COVID-19 (coronavirus disease 2019) vaccines induce immunity through different mechanisms. The aim of this study is to compare the titers of specific antibodies in subjects vaccinated with either the Pfizer-BioNTech COVID-19 vaccine or the Sinopharm vaccine. This prospective observational cohort included Jordanian adults vaccinated with two doses, 21 days apart, of either of the two aforementioned vaccines. Titers were collected 6 weeks after the administration of the second dose. Overall, 288 participants were included, of which 141 were administered the Pfizer-BioNTech vaccine, while 147 were administered the Sinopharm vaccine. Remarkably, 140 (99.3%) of the Pfizer-BioNTech vaccine recipients had positive IgG titers, while 126 (85.7%) of Sinopharm recipients had positive IgG (*p* < 0.001). The mean titer for IgG among Pfizer-BioNTech recipients was 515.5 ± 1143.5 BAU/mL, compared to 170.0 ± 230.0 BAU/mL among Sinopharm subjects (*p* < 0.001). Multivariable regression analysis showed that the Pfizer-BioNTech vaccine positively correlated with positive IgG titers (OR: 25.25; 95% CI: 3.25–196.15; *p* = 0.002), compared with a negative effect of cardiovascular diseases (OR: 0.33; 95% CI: 0.11–0.99; *p* = 0.48) on IgG titers. In conclusion, fully vaccinated recipients of the Pfizer-BioNTech vaccine had superior quantitative efficiency compared to Sinopharm recipients. A booster dose is supported for Sinopharm recipients, or those with chronic immunosuppressive diseases.

## 1. Introduction

COVID-19 (coronavirus disease 2019) continues to endanger socioeconomic and healthcare systems worldwide, with a fluctuating course taking a steep rise after the evolution of the delta variant [1]. A vaccine production race took place globally to counter the growing impact of the pandemic [2,3]. Up to the beginning of August 2021, 4.14 billion doses of vaccine were given worldwide, and 1.14 billion (14.6% of the world’s population) are labeled fully vaccinated [4].

COVID-19 vaccines induce innate and adaptive immunity through different mechanisms. Adaptive immunity, our point of concern, involves an antibody response caused by B cells, which multiply and increase proportionally, leading to the production of specific antibodies that bind to the spike protein to neutralize the viral entry into the cells, thus conferring immunity to severe acute respiratory syndrome coronavirus 2 (SARS-CoV-2) infection [5,6,7]. These antibodies form the ‘immunological memory’, which is the principle of vaccination effectiveness [8]. Serological diagnostic tests are based on the detection of antibodies against nucleocapsid (N) and spike (S) antigens of SARS-CoV-2 [9,10].

At present, two families of vaccines were implemented to counter the spread of COVID-19: mRNA vaccines such as Pfizer-BioNTech, and classic inactivated vaccines such as Sinopharm. The immune surveillance conducted through the measurement of antigen-specific antibody levels helps us to partly justify these vaccines and build an efficient vaccination strategy. Most SARS-CoV-2 vaccines have been developed to induce antibodies that target SARS-CoV-2 spike protein [2], as these antibodies are the most effective type in protecting from the disease and acquired immunity to SARS-CoV-2 is predicted from anti-spike protein receptor-binding domain (anti-S-RBD) immunoglobulin G (IgG) levels [7].

The aim of this study is to compare the titers of specific antibodies in subjects who were vaccinated with the Pfizer-BioNTech vaccine versus those who were vaccinated with Sinopharm. The study gives two vital clues on comparing the efficiency of the previous vaccines and the need for a booster dose added to the regular vaccination regimen of the two doses.

## 2. Materials and Methods

### 2.1. Study Design

This prospective observational cohort was conducted between March and April 2021. The sampled population included Jordanian adults who has been vaccinated with two doses of either Sinopharm or Pfizer-BioNTech COVID-19 vaccines. The two doses were 21 days apart for all included patients, and the enrollment in the study was carried out 6 weeks after the administration of the second dose. It is noteworthy that this observational study was carried out after the voluntary vaccination of the study participants after registering their vaccination preferences on the online vaccination platform provided by the Jordanian ministry of health (MOH) [11]. By the end of the month of April 2021, only 220,594 individuals out of 10.1 million Jordanians (2.184%) had completed their COVID-19 vaccination by administering the two required doses, regardless of the type of vaccine administered (https://ourworldindata.org/covid-vaccinations?country=JOR) (accessed on 30 April 2021). Therefore, by using the abovementioned numbers, with a 95% confidence interval and enrolling a sample size of 288 individuals, the calculated margin of error is 1.69%. Hence, there is a 95% chance that the real value is within ±1.69% of the surveyed value. The study was conducted according to the guidelines of the Declaration of Helsinki and approved by the institutional review board (IRB) committee at the Hashemite University (No. 6/7/2020/2021) on 7 March 2021. All enrolled participants gave a written informed consent prior to participation in this investigation.

### 2.2. Inclusion and Exclusion Criteria

We included Jordanian adults who had been vaccinated with two doses of either the Sinopharm or Pfizer-BioNTech COVID-19 vaccine. We excluded patients with a history of allergies or anaphylaxis, immunocompromised patients, patients on corticosteroids, and patients taking immunosuppressing medications. We also excluded patients who took only one dose of the aforementioned COVID-19 vaccines, as well as those who did not receive the second dose within the recommended interval.

### 2.3. Sampling and Testing

Vitek Immuno Diagnostic Assay Systems (VIDAS^®^, Biomerieux inc., Hazelwood, MO, USA) for SARS-CoV-2 are automated qualitative assays that were used for the detection of immunoglobulin G (IgG) or immunoglobulin M (IgM) specific for SARS-CoV-2 (Cat. No. 423834 and 423833, respectively) in human serum or plasma (lithium heparin) by utilizing the enzyme-linked fluorescent assay (ELFA) technique. These assays combine a two-step sandwich enzyme immunoassay method with a final fluorescence detection (ELFA). The single-use solid-phase receptacle (SPR) coated with the antigen of recombinant SARS-CoV-2 receptor-binding domain (RBD) of the viral spike protein acts as the solid phase, in addition to being the pipetting instrument. The apparatuses perform all of the stages automatically, and the reaction medium is cycled in and out of the SPR several times. The interior of the SPR devices walls is coated with recombinant SARS-CoV-2 antigen. Therefore, after the samples are diluted, the SARS-CoV-2 IgG or IgM are collected into the interior of the SPR device wall. Then, IgG are specifically detected by anti-human IgG, which is labeled with alkaline phosphatase, while IgM is specifically detected similarly by anti-human IgM, also labeled with alkaline phosphatase. Other unbound components are discarded during each of the washing steps. Through the final detection phase, the substrate (4-Methyl-umbelliferyl phosphate) is cycled in and out of the SPR device, which is hydrolyzed into a fluorescent product (4-Methyl-umbelliferone), with the fluorescence being measured at 450 nm. The intensity of this fluorescence is directly proportional to the level of antibody in the studied sample. Thus, the results are automatically calculated based on values stored in the devices’ memory and a tests values are obtained. Fluorescence is measured twice for each studied sample. The first reading is a background reading of the substrate cuvette while the second reading is performed after the SPR device is introduced into the substrate. An index is calculated as a ratio between the relative fluorescence value (RFV) measured in the sample and the RFV obtained for the calibrator, which is humanized recombinant anti-SARS CoV-2 IgG or IgM. The results were first interpreted as positive (index ≥ 1) or negative (index < 1), before being converted into binding antibody units per milliliter (BAU/mL) that correlate with the WHO standard [12].

### 2.4. Statistical Analysis

For statistical analysis, we used the Statistical Package for the Social Sciences (SPSS) version 25.0 (Chicago, IL, USA). After applying descriptive statistics, the data were presented as a number (percent) for categorical variables and mean ± standard deviation for numeric variables. The chi-squared test and Fisher’s exact test were used to compare categorical variables between the aforementioned two vaccines, as well as between those who has positive IgG titers with participants with negative IgG titers. Adjusted residuals were used for post hoc analysis among significant correlations. We used the Kolmogorov–Smirnov test to investigate the normality of distribution of IgG and IgM titers, after which Mann–Whitney U test was used for comparison between the aforementioned groups. Univariable binary logistic regression analysis was applied to predict factors correlated to negativity of IgG titers post-vaccination, and the odds ratio (OR) and a 95% confidence interval of OR (95% CI) were calculated. Variables that were significant in the univariable binary regression model were included in the multivariable binary logistic regression analysis. A correlation with a two-sided *p*-value < 0.05 was considered statistically significant correlation in all aforementioned statistics. 

## 3. Results

Overall, 288 participants were included in this study, of which 141 were administered the Pfizer-BioNTech COVID-19 vaccine, while 147 were administered the Sinopharm vaccine. A comparison between the two groups in demographics, past medical illnesses, and previous history of COVID-19 is illustrated in Table 1. As demonstrated, 140 (99.3%) of those who received the Pfizer-BioNTech vaccine had positive IgG titers, while 126 (85.7%) of those who received the Sinopharm had positive IgG lab results (*p* < 0.001). The mean titer for IgG among those who received Pfizer-BioNTech was 515.5 ± 1143.5 BAU/mL, compared to 170.0 ± 230.0 BAU/mL among Sinopharm subjects (*p* < 0.001). There was no statistical difference in IgM titers between the two groups, either in positivity (*p* = 0.318) or in titer levels (*p* = 0.618). 

When comparing between patients who had positive IgG titers and those with negative titers, we found a significant difference between the two groups in age-group distribution, with 19 (86.4%) of those with negative IgG titers aged more than 60 years (*p* = 0.004), with an average titer of 247.6 ± 255.5 BAU/mL among those aged >60 years, compared to 504.3 ± 1406.4 BAU/mL among those aged between 40 and 60 years, and 299.8 ± 203.3 BAU/mL among those aged between 20 and 40 years (*p* = 0.005). Among participants who received Sinopharm, 21 subjects had negative titers, of which 19 (90.5%) were aged >60, while the remaining 2 (9.5%) participants were aged between 40 and 60 years (*p* = 0.001). Only one participant had negative titer among Pfizer/BioNTech group, who was within the 20–40 years age group. We investigated the effect of medical illnesses on IgG titers, and we found that 12 (54.5%) of those who had negative IgG titers were diabetic (*p* = 0.002), and 9 (40.9%) had cardiovascular or cerebrovascular diseases (*p* < 0.001) (see Table 2).

We further compared between those who had positive and negative IgM titers 6 weeks post-vaccination. No significant differences were found between the two groups in terms of age (*p* = 0.48), gender (*p* = 0.648), BMI (*p* = 0.7), smoking habit (*p* = 0.351), previous history of COVID-19 infection (*p* = 0.233), type of vaccine received (*p* = 0.318), and the positivity of IgG titers (*p* = 0.734), although significant differences were found between the two groups only in the mean IgM titers (*p* < 0.001) and mean IgG titers (*p* = 0.034).

We performed univariable binomial regression analysis for detecting variables influencing the positivity of IgG titers and found a statistically significant correlation with age (*p* = 0.013), type of vaccine received (*p* = 0.002), diabetes mellitus (*p* = 0.003), and cardiovascular diseases (*p* < 0.001); therefore, these factors were included in the multivariable regression analysis (see Table 3). The overall multivariable regression model was significant (*p* < 0.001). We found that the use of the Pfizer-BioNTech vaccine was positively correlated with positive IgG titers (OR: 25.25; 95% CI: 3.25–196.15; *p* = 0.002), compared with a negative effect of cardiovascular diseases (OR: 0.33; 95% CI: 0.11–0.99; *p* = 0.48) on IgG titers. Although diabetes mellitus negatively affected IgG titer positivity, it was not statistically significant (OR: 0.39; 95% CI: 0.14–1.14; *p* = 0.085). 

## 4. Discussion

COVID-19 is still spreading unabated globally, with an urgent need for better understanding of immune response and vaccination strategies [13]. Protection against SARS-CoV-2 can be attained by neutralizing the virus using antibodies (NAbs) [14]. Immunity against SARS-CoV-2 is reflected by the quality, quantity, and duration of the antibody response throughout the course of the disease [15].

Our results suggest a higher quantitative efficiency of the mRNA Pfizer-BioNTech COVID-19 vaccine over the classic Sinopharm, although the exact number that confers immunity is still not clear. For the Pfizer-BioNTech group, 99.3% had positive IgG titers compared to 85.7% of the Sinopharm group. Our results showed a statistically significant difference in IgG antibody titers between both groups, with the Pfizer-BioNTech group mean of 515.5, compared to 170.0 in Sinopharm group. Even though clinical phases of the Sinopharm vaccine have shown a seroconversion rate of 99.3% in the WIV04 SARS-CoV-2 strain compared to 100.0% in HB02 SARS-CoV-2 strain [16], it is noteworthy that this seroconversion rate was based on blood samples collected 14 days after the second dose of the vaccine. Hence, future investigations must include serial titer levels over a broad timeframe in order to have a better understanding of the changes in antibody titers over time. To the best of our knowledge, this is the first study to compare the antibody levels between an mRNA vaccine and an inactivated virus vaccine in the Middle East and North Africa (MENA) region and is among the first studies to compare these vaccines worldwide [17].

Once antibodies are formed, immunological memory can last beyond protective levels for years if initial titers were high enough. In a recent work by Khoury et al. [18], the authors studied the relationship between the neutralization of antibody levels after decay in vitro and acquired protection against COVID-19 and found that the Pfizer-BioNTech vaccine mean neutralization level was higher than the mean convalescent level, which implies more protection. The Sinopharm data were not implemented in the study; however, the Sinovac vaccine, which is thought to work in the same manner, had a mean neutralization level below the convalescent mean. This supports our findings that high titers confer stronger and more durable immunity. Since virus-specific neutralizing antibodies (NAbs) represent the gold standard to evaluate the efficacy of vaccines, we recommend future studies compare the quality of these antibodies through neutralization analysis, taking into consideration previous history of a positive COVID-19 test, whether it was symptomatic or asymptomatic infection, and the severity of the disease [19]. With funding being a major limiting factor for such studies, proper funding is encouraged to enable the inclusion of a larger sample size, allowing comparison of the quality of the neutralizing antibodies based on the aforementioned factors.

Our results showed that old people (>60) had lower titers (with a mean of 247.6) than younger counterparts, which is echoed by a study [20] that showed elderly patients had significantly lower levels of SARS-CoV-2 spike S1 IgG titers than young subjects after receiving the Pfizer-BioNTech vaccine. Although studies have shown that females induce higher titers of IgGs [21,22], no significant difference was found in our study.

Our study collected data on IgG and IgM levels. IgG is the most abundant antibody in human plasma and is the center of our interest because it is largely responsible for long-term immunity after vaccination [23]. In a recent study of 535 plasma samples taken from 173 patients with COVID-19 [24], the median seroconversion time was 12 days and 14 days for IgM and IgG, respectively. IgM remained above the detection threshold for 14 to 21 days from symptom onset, while IgG typically remains above detection level for months to years. This explains why we were not able to detect IgM in our sample as samples were collected 6 weeks following vaccination.

More than 50% of our negative sample had diabetes, and 40% of them had cardiovascular diseases. This is corroborated with multiple studies that discover the effect of diabetes [25,26] and cardiovascular diseases on immunity [27]. Many things can affect the immune response towards COVID-19 and the titer of antibodies [28]. In COVID-19, the disease itself might become an immunosuppressant. Lymphocyte count decreases as the disease progresses [29] and the immunosuppressive IL-10 (Interleukin 10) markedly increases in parallel with disease severity [30].

Only the Pfizer-BioNTech vaccine was significantly associated with positive IgG titers (OR 25.25), and only cardiovascular diseases had a significant negative correlation with IgG (OR 0.33). Previous infection does not guarantee immunity against the virus; a large observational study that compared the level of anti-S-RBD IgG in vaccinated and previously infected patients found that they were significantly lower in recovered patients compared to vaccinated subjects [7]. We realize our study points to mRNA vaccines as better surrogates than classic vaccines, which could encourage vaccine hesitancy due to differential availability of the vaccines [31]. Nevertheless, this should encourage people who have already taken Sinopharm or patients with chronic disease immunosuppression to consider a booster dose [32].

To the best of our knowledge, this is the first study that compares antibody levels in classical Sinopharm and mRNA vaccines. Our study provides important information that could be valuable for booster vaccinations and will help us to build effective vaccination strategies in the future.

This study has some limitations. The samples were collected only once in a specific post-vaccination period (6 weeks). Hence, the study cannot provide information about antibody or immunity decay. The number of patients included in the study was low. Currently, the available SARS-CoV-2 antibody tests lack sufficient sensitivity to allow for accurate estimation of the antibody response [33], although the used diagnostic assay (VIDAS) was shown to have a sensitivity of 88.3% and specificity of 98.4% in a recent study investigating the diagnostic efficiency of fully automated serology assays for SARS-CoV-2 IgG [34]. Furthermore, some cross-reactivity between SARS-CoV-2-specific antibodies and other endemic coronaviruses has been found and can reduce the test reliability [35].

Our study suggests that a booster dose is needed for specific patients, but this suggestion is faced with some challenges. Most studies reporting an immune response after the second dose of the vaccine are limited to the timeframe of the first 3 months following the second dose of the vaccine [36,37]. Other studies have shown a significant antibody titer decline at 3 months compared to the peak response at 3–4 weeks [38], but the sample size was small. Although the aim is to protect vaccinated subjects against severe SARS-CoV-2, many related variants of the virus have already demonstrated many forms of immunity escape [39]. Studies with long-term follow-up are needed to obtain accurate information about the rate of decline in quality and quantity of immune response.

## 5. Conclusions

In conclusion, in fully vaccinated subjects, the Pfizer-BioNTech vaccine has shown superior quantitative efficiency to Sinopharm vaccine. A booster dose is supported for subjects who have had the Sinopharm vaccine, or those with chronic immunosuppressive diseases.

## Figures and Tables

**Table 1:** Comparison between patients receiving Pfizer-BioNTech COVID-19 vaccine and those who received Sinopharm COVID-19 vaccine in terms of demographics, past medical illnesses, and previous history of COVID-19 infection.

Characteristics		Type of Received Vaccine		Total (*n* = 288)	*p*-Value
		Pfizer (*n* = 141)	Sinopharm (*n* = 147)		
Age	20–40 years	19 (13.5)	24 (16.3)	43 (14.9)	0.673
	40–60 years	49 (34.8)	45 (30.6)	94 (32.6)	
	>60 years	73 (51.8)	78 (53.1)	151 (52.4)	
Gender	Male	95 (67.4)	94 (63.9)	189 (65.6)	0.54
	female	46 (32.6)	53 (36.1)	99 (34.4)	
BMI	18.5–24.9 kg/m^2^	16 (11.3)	23 (15.6)	39 (13.5)	0.36
	25–29.9 kg/m^2^	96 (68.1)	95 (64.6)	191 (66.3)	
	30–40 kg/m^2^	29 (20.6)	27 (18.4)	56 (19.4)	
	>40 kg/m^2^	0 (0)	2 (1.4)	2 (0.7)	
Smoking habit	No	91 (64.5)	93 (63.3)	184 (63.9)	0.4
	Yes	46 (32.6)	45 (30.6)	91 (31.6)	
	Ex-smoker	4 (2.8)	9 (6.1)	13 (4.5)	
DM		42 (29.8)	34 (23.1)	76 (26.4)	0.2
HTN		49 (34.8)	54 (36.7)	103 (35.8)	0.726
Pulmonary diseases		4 (2.8)	7 (4.8)	11 (3.8)	0.394
Cardiovascular diseases		11 (7.8)	23 (15.6)	34 (11.8)	0.039
Hyperlipidemia		18 (12.8)	13 (8.8)	31 (10.8)	0.283
Other comorbidities		14 (9.9)	11 (7.5)	25 (8.7)	0.461
Previous history of COVID-19 positive test		3 (2.1)	5 (3.4)	8 (2.8)	0.723
Need for hospital admission	No	3 (2.1)	5 (3.4)	8 (2.8)	0.723
Duration of admission (days)	Not admitted	3 (2.1)	5 (3.4)	8 (2.8)	0.723
Need for ICU admission	No	3 (2.1)	5 (3.4)	8 (2.8)	0.723
Duration of ICU admission (days)	Not admitted	3 (2.1)	5 (3.4)	8 (2.8)	0.723
IgG titers result	Negative	1 (0.7)	21 (14.3)	22 (7.6)	<0.001
	Positive	140 (99.3)	126 (85.7)	266 (92.4)	
IgM titers result	Negative	74 (79.6)	57 (73.1)	131 (76.6)	0.318
	Positive	19 (20.4)	21 (26.9)	40 (23.4)	
IgG titer (BAU/mL)		515.5 ± 1143.5	170.0 ± 230.0	339.2 ± 833.5	<0.001
IgM titer (BAU/mL)		17 ± 29.9	26.1 ± 45.6	21.2 ± 38.1	0.618

BMI: body mass index; DM: diabetes mellitus; HTN: hypertension; COVID-19: coronavirus disease 2019; ICU: intensive care unit; IgG: immunoglobulin G; IgM: immunoglobulin M; BAU/mL: binding antibody units per milliliter. Values are represented as number (percent) and mean ± standard deviation.

**Table 2:** Comparison between patients with positive and negative IgG titers post-vaccination with either BioNTech or Sinopharm COVID-19 vaccine in terms of demographics, past medical illnesses, and previous history of COVID-19 infection.

Characteristics		IgG Titers Result		Total (*n* = 288)	*p*-Value
		Negative (*n* = 22)	Positive (*n* = 266)		
Age	20–40 years	1 (4.5)	42 (15.8)	43 (14.9)	0.004
	40–60 years	2 (9.1)	92 (34.6)	94 (32.6)	
	>60 years	19 (86.4)	132 (49.6)	151 (52.4)	
Gender	Male	13 (59.1)	176 (66.2)	189 (65.6)	0.502
	Female	9 (40.9)	90 (33.8)	99 (34.4)	
BMI	18.5–24.9 kg/m^2^	1 (4.5)	38 (14.3)	39 (13.5)	0.6
	25–29.9 kg/m^2^	16 (72.7)	175 (65.8)	191 (66.3)	
	30–40 kg/m^2^	5 (22.7)	51 (19.2)	56 (19.4)	
	>40 kg/m^2^	0 (0)	2 (0.8)	2 (0.7)	
Smoking habit	No	13 (59.1)	171 (64.3)	184 (63.9)	0.879
	Yes	8 (36.4)	83 (31.2)	91 (31.6)	
	Ex-smoker	1 (4.5)	12 (4.5)	13 (4.5)	
DM		12 (54.5)	64 (24.1)	76 (26.4)	0.002
HTN		11 (50.0)	92 (34.6)	103 (35.8)	0.147
Pulmonary diseases		1 (4.5)	10 (3.8)	11 (3.8)	0.589
Cardiovascular or cerebrovascular diseases		9 (40.9)	25 (9.4)	34 (11.8)	<0.001
Hyperlipidemia		4 (18.2)	27 (10.2)	31 (10.8)	0.274
other comorbidities		4 (18.2)	21 (7.9)	25 (8.7)	0.1
Previous history of COVID-19 positive test		1 (4.5)	7 (2.6)	8 (2.8)	0.475
need for hospital admission	No	1 (4.5)	7 (2.6)	8 (2.8)	0.475
Duration of admission (days)	Not admitted	1 (4.5)	7 (2.6)	8 (2.8)	0.475
Need for ICU admission	No	1 (4.5)	7 (2.6)	8 (2.8)	0.475
Duration of ICU admission (days)	Not admitted	1 (4.5)	7 (2.6)	8 (2.8)	0.475
Type of received vaccine	Pfizer-BioNTech	1 (4.5)	140 (52.6)	141 (49)	<0.001
	Sinopharm	21 (95.5)	126 (47.4)	147 (51)	
IgM result	Negative	10 (83.3)	121 (76.1)	131 (76.6)	0.734
	Positive	2 (16.7)	38 (23.9)	40 (23.4)	

BMI: body mass index; DM: diabetes mellitus; HTN: hypertension; COVID-19: coronavirus disease 2019; ICU: intensive care unit; IgG: immunoglobulin G; IgM: immunoglobulin M; BAU/mL: binding antibody units per milliliter. Values are represented as number (percent) and mean ± standard deviation.

**Table 3:** Univariable and multivariable binomial regression analysis for factor effects on COVID-19 IgG titers positivity among vaccinated individuals.

Characteristics	Univariable				Multivariable			
	OR	95% C.I. for OR		*p*-Value	OR	95% C.I. for OR		*p*-Value
		Lower	Upper			Lower	Upper	
Age				0.013				0.107
Age (20–40 years)	6.045	0.786	46.523	0.084	3.426	0.398	29.502	0.262
Age (40–60 years)	6.621	1.506	29.120	0.012	4.621	0.975	21.908	0.054
Gender (male)	1.354	0.558	3.287	0.503	-	-	-	-
BMI				0.684	-	-	-	-
BMI (25–29.9 kg/m^2^)	0.288	0.037	2.237	0.234	-	-	-	-
BMI (30–39.9 kg/m^2^)	0.268	0.030	2.393	0.239	-	-	-	-
BMI (>40 kg/m^2^)	42,512,495.865	0.000		1.000	-	-	-	-
Smoking habit				0.880	-	-	-	-
Smoking habit (yes)	0.789	0.315	1.977	0.613	-	-	-	-
Smoking habit (ex-smoker)	0.912	0.110	7.574	0.932	-	-	-	-
Type of received vaccine (Pfizer-BioNTech)	23.333	3.094	175.978	0.002	25.255	3.252	196.151	0.002
Previous history of COVID 19 positive test	0.568	0.067	4.833	0.604	-	-	-	-
DM	0.264	0.109	0.640	0.003	0.393	0.136	1.137	0.085
HTN	0.529	0.221	1.266	0.153	-	-	-	-
Cardiovascular disease	0.150	0.058	0.385	<0.001	0.328	0.108	0.992	0.048
Hyperlipidemia	0.508	0.160	1.612	0.251	-	-	-	-
Pulmonary disease	0.820	0.100	6.720	0.854	-	-	-	-

BMI: body mass index; COVID-19: coronavirus disease 2019; DM: diabetes mellitus; HTN: hypertension; OR: odds ratio; 95% CI: 95% confidence interval. We used the age group “>60 years” and the BMI group “18.5–24.9 kg/m^2^” as reference standard for all comparisons.

## Data Availability

The data presented in this study are available upon request from the corresponding author.
